# Temporal Changes in Pediatric Influenza Test Positivity Across COVID-19 Public Health Policy Phases: A Five-Year Retrospective Cohort Study

**DOI:** 10.7759/cureus.113386

**Published:** 2026-07-26

**Authors:** Yunus Nas, Suveyda Gozukucuk, Basri Cakiroglu

**Affiliations:** 1 School of Health Sciences, Doğuş University, Istanbul, TUR; 2 Department of Infectious Diseases and Clinical Microbiology, Hisar Hospital Intercontinental, Istanbul, TUR; 3 Department of Urology, Üsküdar University, Istanbul, TUR

**Keywords:** children, covid-19, epidemiology, influenza, non-pharmaceutical interventions, respiratory viruses, retrospective cohort

## Abstract

Background

The COVID-19 pandemic coincided with substantial changes in respiratory virus circulation, healthcare utilization, and diagnostic testing. Although population-level influenza activity declined markedly in many settings during periods of stringent public health restrictions, patterns among children undergoing clinical testing remain less clearly understood. This study compared pediatric influenza test positivity across pre-pandemic, restriction-dominant pandemic, and post-restriction periods.

Methods

This retrospective cohort study included 18,035 children who underwent influenza A/B antigen testing at the Department of Pediatrics, Hisar Intercontinental Hospital, Istanbul, Türkiye, between January 2018 and May 2023. The study period was divided into the pre-pandemic (January 2018-February 2020), restriction-dominant pandemic (March 2020-June 2021), and post-restriction (July 2021-May 2023) periods. Influenza A and B antigens were detected in nasopharyngeal swab specimens using the GenBody Influenza A/B Ag rapid immunochromatographic assay. Tests performed for the same patient within 14 days were considered part of the same illness episode, and only the first eligible test was included. Influenza test positivity was compared across periods and according to age, sex, influenza type, and seasonality. Multivariable logistic regression was used to evaluate factors independently associated with influenza positivity.

Results

Of the 18,035 children, 6,691 were tested during the pre-pandemic period, 3,375 during the restriction-dominant period, and 7,969 during the post-restriction period. Influenza was detected in 1,699 (25.4%), 969 (28.7%), and 1,937 (24.3%) children, respectively (P<0.001). Although the proportion of positive tests was highest during the restriction-dominant period, both the testing volume and absolute number of detected cases were lower than before the pandemic. Influenza A positivity increased from 17.1% during the restriction-dominant period to 20.0% after restrictions were relaxed, whereas influenza B positivity declined from 11.6% to 4.4%. Seasonal positivity also varied substantially across the study periods. After adjustment for demographic characteristics, the epidemiological period remained independently associated with influenza test positivity.

Conclusions

Temporal changes in pediatric influenza test positivity coincided with changes in COVID-19 public health measures. The higher positivity among tested children during the restriction-dominant period occurred in the context of substantially reduced testing and should not be interpreted as increased population-level influenza circulation. Changes in healthcare utilization, testing behavior, and the composition of the tested population should be considered when interpreting these findings.

## Introduction

Influenza remains a major cause of acute respiratory illness in children and continues to impose a substantial global health burden despite the availability of effective vaccines and antiviral treatments. Children contribute substantially to influenza transmission because of their high susceptibility, prolonged viral shedding, and frequent close contact within households, childcare facilities, and schools. Seasonal influenza therefore results in considerable pediatric morbidity, outpatient visits, hospitalizations, school absenteeism, and healthcare expenditures. Continuous surveillance is essential for characterizing temporal patterns, guiding vaccination strategies, and informing healthcare preparedness [1-4].

According to the World Health Organization’s 2025 estimates, seasonal influenza causes approximately one billion infections annually worldwide, including 3-5 million cases of severe illness and 290,000-650,000 respiratory deaths [5]. The emergence of coronavirus disease 2019 (COVID-19) profoundly altered the epidemiology of respiratory viruses. To limit the transmission of severe acute respiratory syndrome coronavirus 2 (SARS-CoV-2), countries implemented measures including masking, physical distancing, school closures, travel restrictions, curfews, enhanced hand hygiene, and limitations on social gatherings. Although primarily intended to control SARS-CoV-2, these measures coincided with historically low population-level influenza activity in many countries [6-9].

As restrictions were relaxed, influenza activity re-emerged in many settings, frequently with altered timing and seasonal patterns. Several mechanisms may have contributed to these changes. Prolonged suppression of influenza circulation may have reduced population-level immune experience, while the restoration of routine social interactions increased opportunities for transmission [8,10,11]. Viral interference among co-circulating respiratory viruses may also have temporarily modified susceptibility to influenza through innate antiviral responses [2]. However, SARS-CoV-2 coinfection and sequential viral infections were not systematically evaluated in most retrospective datasets, limiting direct assessment of this mechanism.

Changes in healthcare utilization and testing behavior are also important when interpreting pandemic-period findings. Children with mild respiratory symptoms may have been less likely to attend healthcare facilities, whereas influenza testing may have become concentrated among patients with greater symptom severity or stronger clinical suspicion. Consequently, positivity among tested children may not directly correspond to population-level influenza incidence or total disease burden. Differences in healthcare-seeking behavior, testing volume, vaccination coverage, demographic characteristics, and public health policies may partly explain the substantial variation observed among countries [8,12].

Several pediatric studies from different countries have documented marked changes in influenza circulation during and after the COVID-19 pandemic [13-16]. Nevertheless, multinational or pooled analyses may obscure country-specific differences in the timing and intensity of restrictions, healthcare utilization, testing practices, and relaxation policies. Moreover, some studies used binary pre-pandemic versus pandemic comparisons, which may not capture phase-dependent changes as restrictions were progressively withdrawn. The present study was designed to complement, rather than duplicate, this international evidence by evaluating three predefined epidemiological periods within a single healthcare system, using consistent specimen collection and laboratory testing procedures across consecutive influenza seasons. This approach enables assessment of local changes in influenza test positivity while minimizing between-center variation in diagnostic practice.

Türkiye provides a valuable setting for examining these epidemiological changes because comprehensive public health measures were implemented nationwide during the COVID-19 pandemic and subsequently relaxed through officially defined stages of gradual normalization [17-20]. The first stage began on May 17, 2021, and a second stage was implemented during June 2021 [17,18]. Because substantial nationwide restrictions remained in effect throughout this transitional interval, May 17-June 30, 2021, was retained within the restriction-dominant pandemic period in the predefined analysis. The post-restriction period began on July 1, 2021, when broader nationwide restrictions were progressively lifted. The national policy transitions and their relationship to the analytical periods are summarized in Appendices.

The primary objective of this study was to compare pediatric influenza test positivity across the pre-pandemic, restriction-dominant pandemic, and post-restriction periods. The secondary objectives were to characterize temporal and seasonal patterns of influenza A and B and identify demographic factors independently associated with influenza positivity. We hypothesized that overall and type-specific influenza test positivity would differ across the three epidemiological periods

## Materials and methods

Study design and setting

This retrospective cohort study was conducted at the Department of Pediatrics, Hisar Intercontinental Hospital, Istanbul, Türkiye. Electronic medical records of pediatric patients who underwent influenza A/B antigen testing between January 1, 2018, and May 31, 2023, were reviewed. The study evaluated temporal differences in pediatric influenza test positivity across three predefined epidemiological periods corresponding to changes in national COVID-19 public health policies.

The study protocol was approved by the Institutional Review Board of Hisar Intercontinental Hospital (Approval No. 23-46/2026). Because anonymized, routinely collected clinical data were analyzed retrospectively, the requirement for informed consent was waived by the Institutional Review Board. The study was conducted in accordance with the principles of the Declaration of Helsinki.

Study population

Children aged 0-18 years who presented to the pediatric outpatient clinics or emergency department with symptoms suggestive of an acute respiratory tract infection and underwent influenza A/B antigen testing during the study period were eligible. Influenza testing was requested at the treating physician’s discretion based on clinical suspicion rather than through a universal screening protocol.

Patients were excluded if demographic information was missing, the influenza test result was invalid or inconclusive, or the record represented a duplicate test within the same illness episode. An illness episode was operationally defined using a 14-day interval. Tests performed for the same patient within 14 days of the initial eligible test were considered part of the same episode, and only the first test was included. A test performed more than 14 days after the preceding eligible test was considered a new illness episode.

Because this was a retrospective census-based cohort study, no a priori sample-size calculation was performed. All patients meeting the eligibility criteria during the predefined study period were included.

Definition of study periods

Patients were classified into three epidemiological periods according to the national COVID-19 public health policy timeline in Türkiye. The pre-pandemic period extended from January 1, 2018, to February 29, 2020. The restriction-dominant pandemic period extended from March 1, 2020, to June 30, 2021, and encompassed nationwide measures including school closures, mandatory masking, physical distancing, travel restrictions, curfews, and limitations on social gatherings.

An officially defined gradual-normalization process began on May 17, 2021, with a second stage introduced in June 2021. Because substantial nationwide restrictions remained in effect during this transitional interval, May 17-June 30, 2021, was retained within the restriction-dominant period for the predefined analysis. The post-restriction period extended from July 1, 2021, to May 31, 2023, during which most nationwide restrictions were progressively withdrawn, and routine social and educational activities resumed. The national policy transitions and their relationship to the analytical periods are summarized in Appendix A.

Influenza testing

Nasopharyngeal swab specimens were collected by trained healthcare professionals according to standardized clinical procedures. Influenza A and B antigens were detected using the GenBody Influenza A/B Ag rapid test (GenBody Inc., Cheonan-si, Republic of Korea), a qualitative immunochromatographic assay used consistently throughout the study period. Testing and interpretation were performed by experienced laboratory personnel according to the manufacturer’s instructions.

The manufacturer reports sensitivity and specificity of 100% and 96.22%, respectively, for influenza A and 98.39% and 94.76%, respectively, for influenza B. Manufacturer-reported positive and negative predictive values were unavailable. Because predictive values depend on disease prevalence and rapid-test findings were not routinely verified against reverse-transcription polymerase chain reaction or viral culture, study-specific positive and negative predictive values could not be calculated. A positive influenza A and/or influenza B antigen result was classified as laboratory-detected influenza for the purposes of this study.

Data collection

Demographic and laboratory data were extracted from the hospital electronic medical record system. The collected variables included age, sex, date of presentation, epidemiological period, influenza test result, and influenza type. Patients were categorized into four predefined age groups: <2, 2-5, 6-12, and 13-18 years.

Monthly numbers of tested and influenza-positive children were calculated alongside monthly positivity rates to evaluate changes in testing volume, temporal patterns, and seasonality. Information on symptom severity, the reason for requesting testing, influenza vaccination status, comorbidities, childcare or school attendance, previous influenza infection, and SARS-CoV-2 coinfection was not consistently available and could not be included in the analysis.

**Figure 1:**
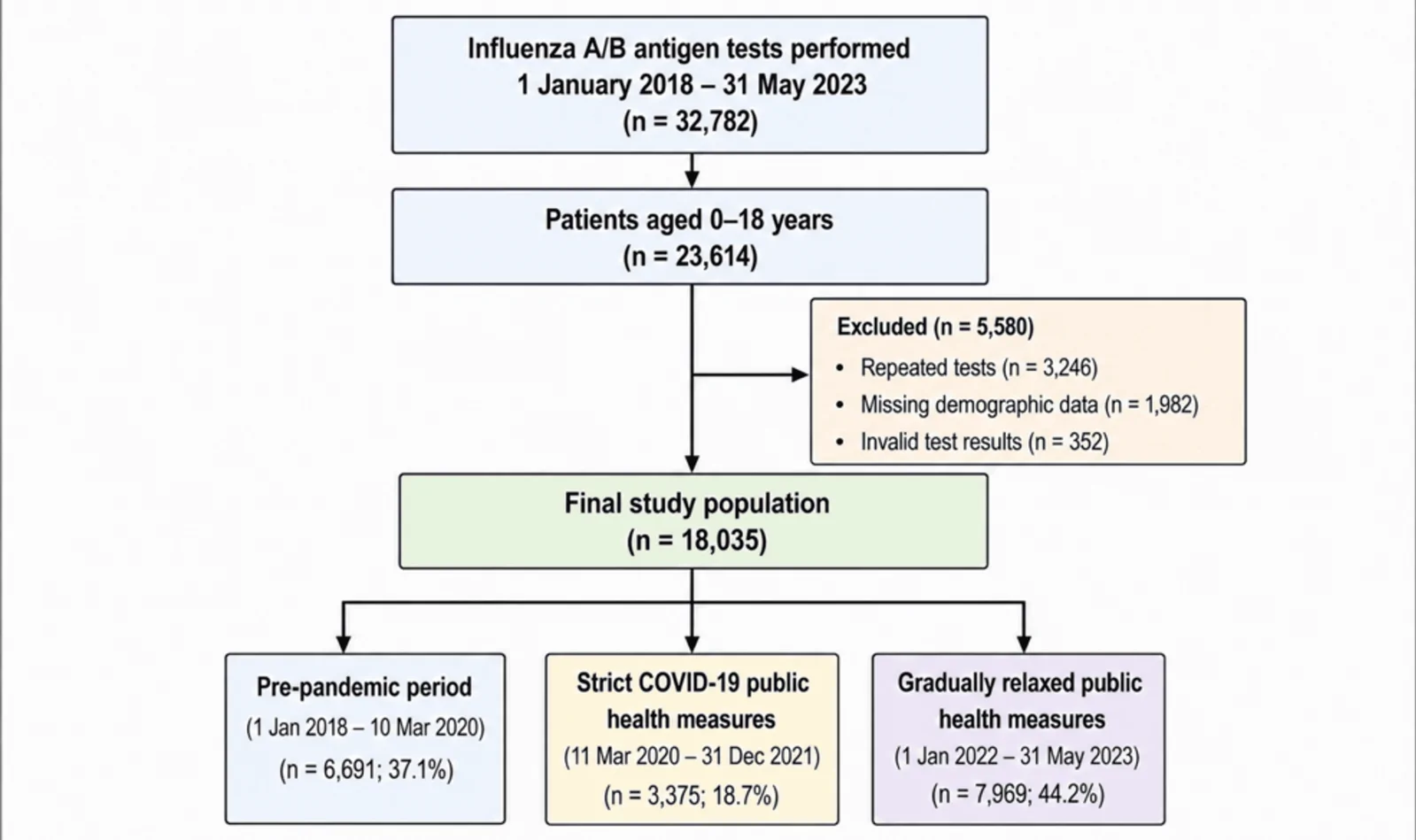
Study flow diagram

Figure 1 shows the study flow diagram.

Outcome measures

The primary outcome was influenza test positivity, defined as a positive rapid antigen result for influenza A and/or influenza B. Secondary outcomes included differences in overall test positivity across the three epidemiological periods, type-specific patterns of influenza A and B, age- and sex-specific positivity, monthly and seasonal patterns, and demographic factors independently associated with influenza positivity.

These outcomes represent influenza positivity among children selected for testing and were not considered measures of population-level influenza incidence or total community disease burden.

Statistical analysis

Continuous variables were assessed for normality using the Shapiro-Wilk test. Normally distributed variables are presented as mean ± standard deviation and were compared using the independent-samples t test or one-way analysis of variance, as appropriate. Non-normally distributed variables are presented as median and interquartile range and were compared using the Mann-Whitney U test or Kruskal-Wallis test, as appropriate.

Categorical variables are presented as frequencies and percentages and were compared using Pearson’s chi-square test or Fisher’s exact test. Period-specific analyses included the absolute number of children tested, the absolute number with positive results, and the corresponding positivity rate. Monthly testing volumes and positive-case counts were evaluated descriptively alongside positivity rates to support interpretation of temporal changes.

Multivariable binary logistic regression was performed to identify factors independently associated with influenza positivity. Age group, sex, and epidemiological period were included as prespecified covariates. Adjusted odds ratios and 95% confidence intervals were calculated, and reference categories were identified explicitly in the regression table.

Multicollinearity was assessed using variance inflation factors. Model calibration was evaluated using the Hosmer-Lemeshow goodness-of-fit test, and discrimination was assessed using the area under the receiver operating characteristic curve. Residual patterns and influential observations were also examined.

The extent of missing data was assessed for each variable. No imputation was performed, and analyses were conducted using complete cases. All statistical tests were two-sided, and P<0.05 was considered statistically significant.

Statistical analyses were performed using IBM SPSS Statistics for Windows, version 26.0 (IBM Corp., Armonk, NY, USA). Time-series figures were generated using R version (exact version) (R Foundation for Statistical Computing, Vienna, Austria).

## Results

Study population

A total of 18,035 pediatric patients who underwent influenza A/B antigen testing between January 1, 2018, and May 31, 2023, were included in the final analysis. The mean age of the study population was 5.65 ± 3.94 years, and the median age was 5 years (interquartile range (IQR), 3-8 years). Overall, 9,932 patients (55.1%) were male and 8,103 (44.9%) were female. Children aged 2-5 years constituted the largest age group, followed by those aged 6-12 years, whereas adolescents aged 13-18 years represented the smallest subgroup (Table 1).

According to the predefined epidemiological periods, 6,691 patients (37.1%) were evaluated during the pre-pandemic period, 3,375 (18.7%) during the period of strict COVID-19 public health measures, and 7,969 (44.2%) during the subsequent period of gradually relaxed public health measures (Table 1).

**Table 1:** Baseline characteristics of the study population NPI: non-pharmaceutical intervention

Baseline characteristics	Overall (n = 18,035)
Age, years	
Mean ± SD	5.65 ± 3.94
Median (IQR)	5 (3–8)
Sex	
Male	9,932 (55.1%)
Female	8,103 (44.9%)
Age group, years	
<2	2,344 (13.0%)
2–5	7,999 (44.4%)
6–12	6,308 (35.0%)
13–18	1,383 (7.7%)
Study period	
Pre-pandemic	6,691 (37.1%)
Strict NPI	3,375 (18.7%)
Post-restriction	7,969 (44.2%)
Influenza test results	
Influenza A positive	3,267 (18.1%)
Influenza B positive	1,337 (7.4%)
Overall influenza positive (A and/or B)	4,604 (25.5%)
Influenza negative	13,431 (74.5%)

Influenza positivity across epidemiological periods

Overall, 4,604 of 18,035 children (25.5%) tested positive for influenza A and/or influenza B, whereas 13,431 (74.5%) had negative test results.

Influenza positivity differed significantly across the three epidemiological periods (Table 2). Overall influenza positivity was 25.4% during the pre-pandemic period, increased to 28.7% during the period of strict COVID-19 public health measures, and subsequently declined to 24.3% during the period of gradually relaxed public health measures (P<0.001).

**Table 2:** Influenza positivity according to epidemiological period NPI: non-pharmaceutical intervention

Variable	Pre-pandemic (n=6,691)	Strict NPI (n=3,375)	Post-restriction (n=7,969)	Statistical test	Test statistic	P value
Influenza A positive, n (%)	1,101 (16.5)	576 (17.1)	1,590 (20.0)	Pearson χ²	33.07	<0.001
Influenza B positive, n (%)	598 (8.9)	393 (11.6)	347 (4.4)	Pearson χ²	219.16	<0.001
Overall influenza positive (A and/or B), n (%)	1,699 (25.4)	969 (28.7)	1,937 (24.3)	Pearson χ²	24.30	<0.001
Influenza negative, n (%)	4,992 (74.6)	2,406 (71.3)	6,032 (75.7)	Pearson χ²	24.30	<0.001

The distribution of influenza virus types also changed significantly across the study periods. Influenza A positivity increased from 16.5% during the pre-pandemic period to 17.1% during the period of strict public health measures and reached its highest level (20.0%) during the period of gradually relaxed public health measures (P<0.001). In contrast, influenza B positivity followed a different temporal pattern, increasing from 8.9% during the pre-pandemic period to 11.6% during the period of strict public health measures before declining to 4.4% during the period of gradually relaxed public health measures (P<0.001).

Age-specific influenza positivity

Age-stratified analyses demonstrated significant differences in influenza positivity across the three epidemiological periods (Table 3). Among children younger than 2 years, influenza positivity declined markedly during the period of strict COVID-19 public health measures (6.2%) compared with the pre-pandemic period (14.9%) and subsequently increased during the period of gradually relaxed public health measures (13.0%) (P<0.001).

**Table 3:** Age-specific influenza positivity according to epidemiological period NPI: non-pharmaceutical intervention

Age group (years)	Pre-pandemic: n/N (%)	Strict NPI: n/N (%)	Post-restriction: n/N (%)	p value
<2	200/1,346 (14.9)	8/129 (6.2)	113/869 (13.0)	<0.001
2–5	1,217/4,537 (26.8)	42/256 (16.4)	682/3,206 (21.3)	<0.001
6–12	919/2,984 (30.8)	62/227 (27.3)	872/3,097 (28.2)	0.315*
13–18	175/537 (32.6)	11/49 (22.4)	270/797 (33.9)	0.118*

A similar pattern was observed among children aged 2-5 years, in whom influenza positivity decreased from 26.8% during the pre-pandemic period to 16.4% during the period of strict public health measures and subsequently increased to 21.3% during the period of gradually relaxed public health measures (P<0.001).

In contrast, influenza positivity among school-aged children (6-12 years) and adolescents (13-18 years) remained relatively stable across the three epidemiological periods, with no statistically significant differences observed (P=0.315 and P=0.118, respectively).

Factors associated with influenza positivity

Multivariable logistic regression analysis was performed to identify factors independently associated with influenza positivity (Table 4). Increasing age was independently associated with higher odds of influenza positivity. Compared with children younger than 2 years, the likelihood of influenza positivity increased progressively among children aged 2-5 years, 6-12 years, and 13-18 years.

**Table 4:** Multivariable logistic regression analysis of factors associated with influenza positivity NPI: non-pharmaceutical intervention

Multivariable logistic regression analysis	Adjusted OR (95% CI)	p value
Age group		
<2 years	Reference	—
2–5 years	2.01 (1.77–2.29)	<0.001
6–12 years	2.66 (2.34–3.03)	<0.001
13–18 years	3.20 (2.72–3.77)	<0.001
Sex		
Female	Reference	—
Male	1.01 (0.94–1.08)	0.85
Study period		
Pre-pandemic	Reference	—
Strict NPI	0.63 (0.51–0.77)	<0.001
Post-restriction	0.83 (0.78–0.89)	<0.001

After adjustment for age and sex, the epidemiological period remained independently associated with influenza positivity. Compared with the pre-pandemic period, influenza positivity differed significantly during both the period of strict COVID-19 public health measures and the subsequent period during which these public health measures were gradually relaxed. In contrast, sex was not independently associated with influenza positivity.

Temporal trends in influenza activity

**Figure 2:**
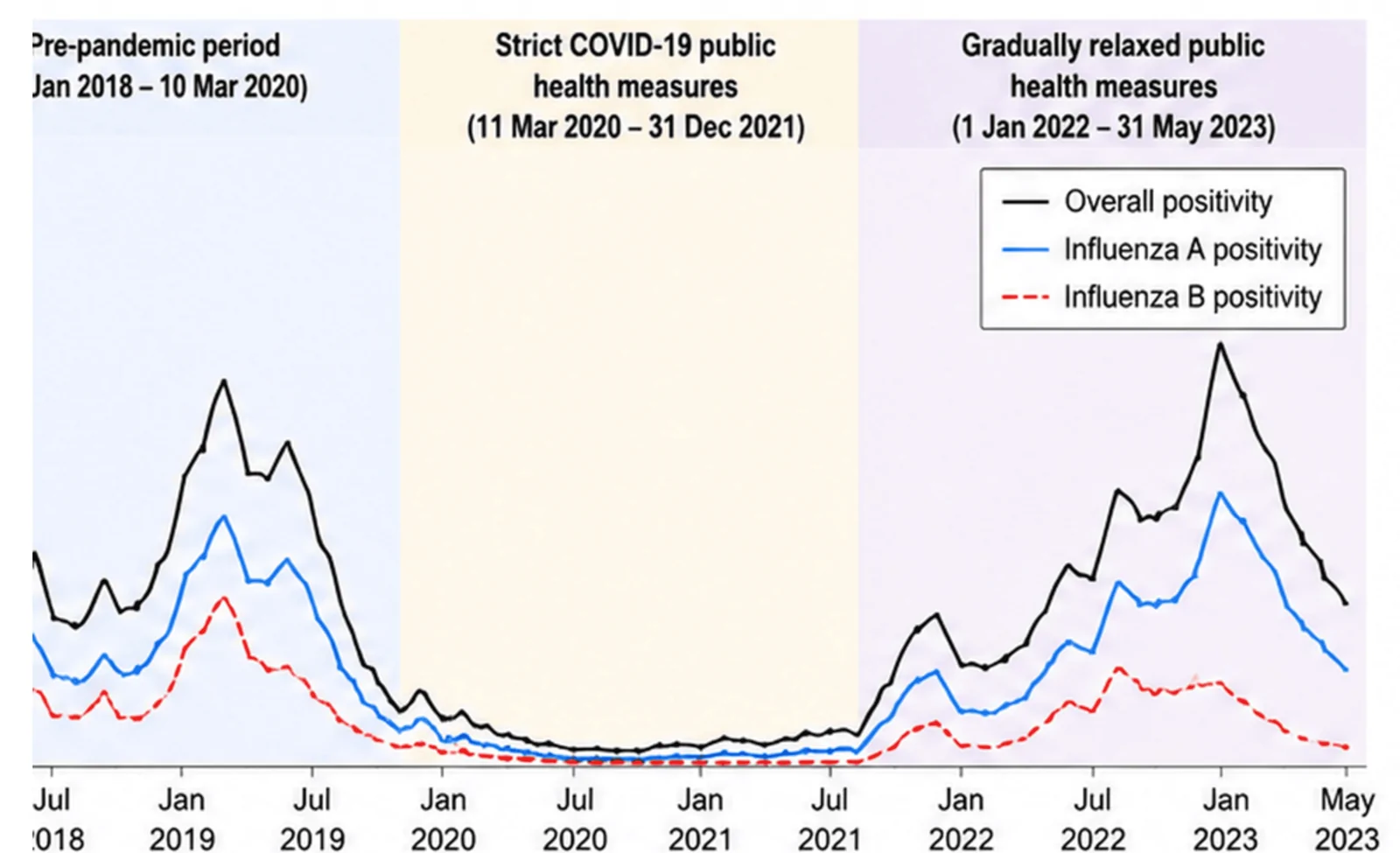
Monthly influenza A, influenza B, and overall influenza positivity throughout the study period

**Figure 3:**
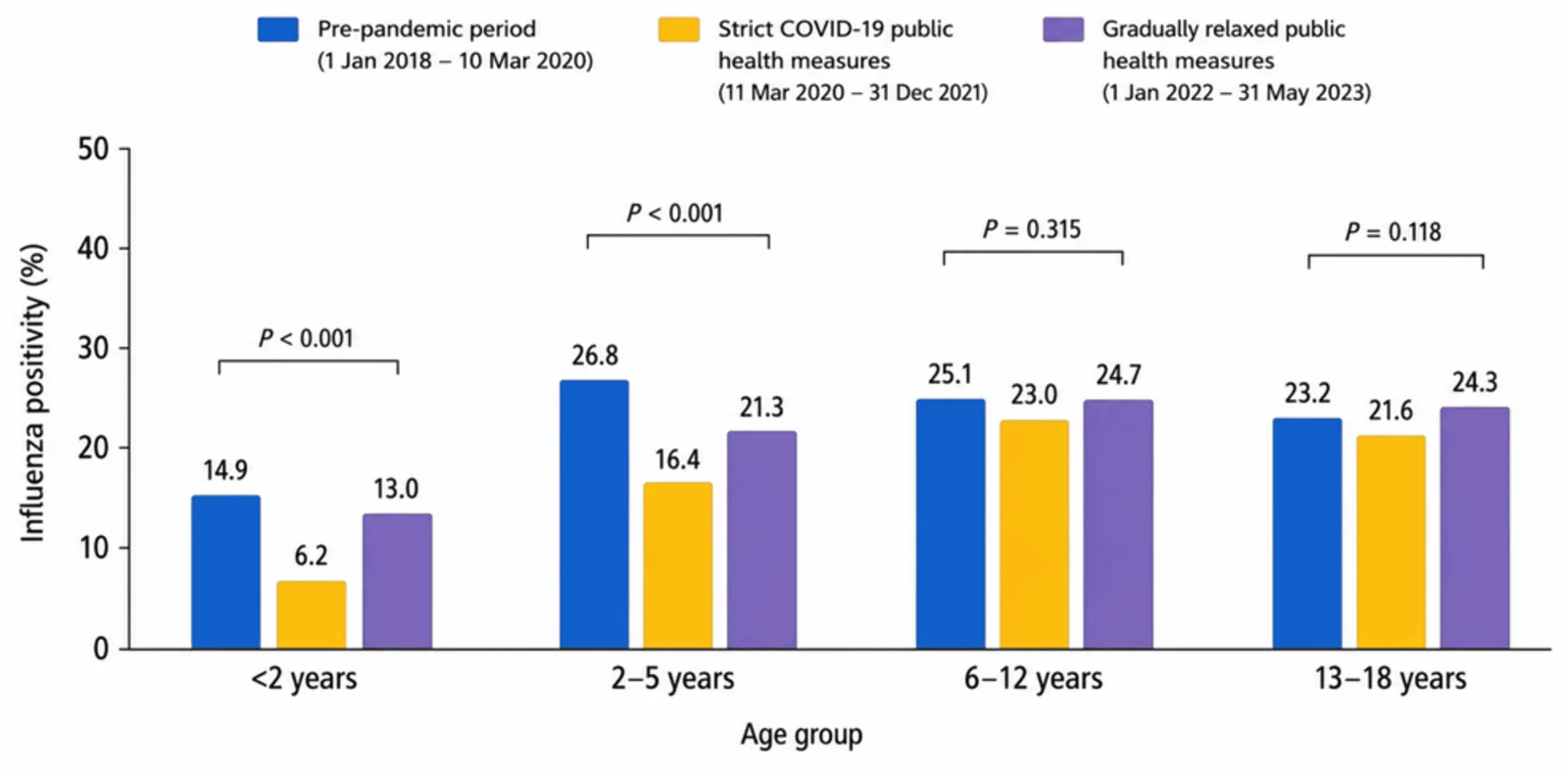
Age-specific influenza positivity across the three epidemiological periods

Monthly analyses demonstrated marked temporal fluctuations in influenza activity throughout the study period (Figures 2, 3). Influenza circulation varied considerably across the three epidemiological periods, with distinct changes observed during the period of strict COVID-19 public health measures and the subsequent period of gradually relaxed public health measures. Influenza A became the predominant circulating virus during the latter period, whereas influenza B declined substantially compared with the earlier periods. Seasonal influenza patterns gradually re-emerged as public health measures were progressively relaxed; however, both the timing and magnitude of seasonal peaks differed from those observed during the pre-pandemic period.

## Discussion

The present study provides a comprehensive evaluation of changes in pediatric influenza epidemiology across three distinct epidemiological periods defined by the implementation and subsequent gradual relaxation of COVID-19 public health measures in a large tertiary-care cohort. Using more than 18,000 influenza A/B antigen tests performed over a seven-year period, we demonstrated that influenza circulation varied substantially across these periods, reflecting marked changes in viral transmission dynamics during the COVID-19 pandemic. Four principal findings emerged from this study. First, influenza positivity differed significantly across the three epidemiological periods, highlighting the close association between changing public health measures and influenza circulation. Second, influenza A and influenza B exhibited distinct temporal patterns, with influenza A becoming the predominant circulating virus during the period of gradually relaxed public health measures, whereas influenza B declined considerably. Third, the impact of changing epidemiological conditions differed according to age, with children younger than 6 years demonstrating the greatest fluctuations in influenza positivity. Finally, multivariable analysis confirmed that both age and epidemiological period were independently associated with influenza positivity, whereas sex was not.

These findings are consistent with accumulating international evidence demonstrating that the COVID-19 pandemic fundamentally reshaped the epidemiology of seasonal respiratory viruses. During periods of strict public health measures, including school closures, universal masking, physical distancing, travel restrictions, and enhanced hygiene practices, many countries experienced an unprecedented disruption of influenza circulation. Large surveillance networks from Europe, North America, Asia, and Australia reported historically low influenza activity throughout 2020 and much of 2021, emphasizing that interventions introduced to reduce SARS-CoV-2 transmission also profoundly affected the spread of other respiratory viruses [7,8,13,14]. Historical evidence from the 1918 influenza pandemic likewise indicates that public health interventions were associated with regional differences in epidemic growth [21]. Recent evidence syntheses have confirmed that pandemic-era epidemiological changes occurred across multiple infectious diseases, although their magnitude differed according to local public health policies, healthcare utilization, vaccination coverage, and population behavior [22].

As public health measures were progressively relaxed, influenza activity gradually re-emerged; however, recovery was neither immediate nor uniform across geographical regions. Recent studies have demonstrated that respiratory pathogens returned asynchronously following relaxation of COVID-19 control measures, suggesting that the re-establishment of circulation depends not only on the removal of public health restrictions but also on pathogen-specific biological characteristics, population immunity, climatic conditions, and changing patterns of social interaction [10,11,22-25]. Consistent with these observations, pediatric surveillance studies have documented substantial rebounds in influenza activity following the resumption of routine school attendance, increased domestic and international travel, and normalization of interpersonal contact [13-16,23]. Our findings from Türkiye closely parallel these international observations while extending current knowledge by distinguishing between the period of strict public health measures and the subsequent period during which these measures were gradually relaxed. Rather than simply comparing pre-pandemic and pandemic periods, our study demonstrates that pediatric influenza epidemiology evolved dynamically across successive phases of changing public health policies.

One of the most notable findings of the present study was the differential behavior of influenza A and influenza B. Influenza A became increasingly predominant during the period of gradually relaxed public health measures, whereas influenza B declined substantially compared with earlier periods. Similar type-specific differences have been reported in several countries following the COVID-19 pandemic and likely reflect differences in viral evolution, transmissibility, host immunity, and ecological competition among circulating respiratory viruses [11,13-16,23]. Influenza B transmission is generally more dependent on sustained circulation within school-aged children, making it particularly susceptible to prolonged interruptions in social mixing. In contrast, influenza A appears to recover more rapidly following restoration of population mobility and interpersonal contact, consistent with observations from recent international surveillance studies [11,13-16,23].

An additional important observation was the marked age-related heterogeneity in influenza positivity. Children younger than six years experienced the largest fluctuations across the three epidemiological periods, whereas influenza positivity among older children and adolescents remained comparatively stable. Younger children generally possess limited pre-existing immunity to influenza viruses and rely heavily on repeated community exposure to develop protective immune responses. Consequently, prolonged periods of reduced viral circulation may disproportionately influence influenza susceptibility in this age group. Furthermore, reopening of daycare centers and kindergartens likely restored opportunities for close interpersonal contact earlier than in other community settings, contributing to the observed epidemiological changes. Similar age-specific patterns have been described in recent pediatric surveillance studies from Europe, North America, the Middle East, and East Asia [13-16].

Study limitations and future directions

Several limitations of this study should be acknowledged. First, this was a retrospective, single-center study, which may limit the generalizability of the findings to other geographic regions or healthcare settings with different influenza epidemiology, healthcare-seeking behaviors, or public health policies. Second, influenza diagnosis was based on rapid antigen testing rather than molecular assays. Although rapid antigen tests are widely used in routine clinical practice and provide timely diagnostic information, their sensitivity is lower than that of reverse transcription polymerase chain reaction (RT-PCR), potentially leading to an underestimation of the true burden of influenza infection. Third, information on influenza vaccination status, underlying comorbidities, socioeconomic characteristics, and prior influenza infection was not consistently available and therefore could not be incorporated into the analyses. These factors may have influenced individual susceptibility to influenza and healthcare utilization during different phases of the pandemic. Finally, as an observational study, our findings demonstrate temporal associations between changing public health measures and influenza epidemiology but do not establish causal relationships.

Despite these limitations, the study has several important strengths, including the large sample size, seven-year observation period, standardized diagnostic approach, and evaluation of influenza epidemiology across three distinct phases of changing public health measures within the same healthcare system. These features enabled a comprehensive assessment of temporal changes while minimizing variability related to differences in diagnostic practices and healthcare infrastructure.

Future multicenter prospective studies incorporating molecular diagnostic methods, influenza vaccination history, viral genomic surveillance, and co-circulating respiratory pathogens are warranted to further elucidate the mechanisms underlying changes in influenza transmission following large-scale public health interventions. In addition, integrating epidemiological surveillance with viral sequencing, immunological assessments, and mathematical transmission modeling may improve understanding of how future public health measures influence the circulation, seasonality, and subtype dynamics of influenza and other respiratory viruses in pediatric populations.

## Conclusions

In conclusion, this five-year retrospective cohort study identified significant temporal differences in pediatric influenza test positivity across the pre-pandemic, restriction-dominant pandemic, and post-restriction periods. Influenza A and B exhibited distinct period-specific patterns, and age was independently associated with influenza positivity. The higher proportion of positive tests during the restriction-dominant period occurred alongside substantially reduced testing volume and should not be interpreted as increased population-level influenza circulation. Changes in healthcare utilization, testing behavior, and the characteristics of the tested population should be considered when interpreting these findings. Continued age- and type-specific influenza surveillance may support pediatric vaccination planning and healthcare preparedness during future disruptions in respiratory virus epidemiology.

## Appendices

**Table 5:** National COVID-19 public health policy timeline and study-period classification in Türkiye

Period	National policy phase	Principal measures	Study classification
January 1, 2018–March 10, 2020	Pre-pandemic	Routine schooling, travel, healthcare utilization, and social activities; no COVID-19-related restrictions	Pre-pandemic period
March 11, 2020–April 13, 2021	Initial response and intensified restrictions	School closures, distance education, mandatory masking, limitations on gatherings, travel restrictions, physical distancing, and intermittent curfews	Strict-NPI period
April 14–May 16, 2021	Partial and full lockdown	Strengthened curfews, limitations on intercity travel, closure or restriction of nonessential activities, and substantially reduced in-person social contact	Strict-NPI period
May 17–May 31, 2021	First stage of gradual normalization	Partial easing of curfews, travel limitations, and restrictions on commercial and social activities	Strict-NPI period*
June 1–June 30, 2021	Second stage of gradual normalization	Further easing of curfews, intercity travel restrictions, and limitations on workplaces and gatherings	Strict-NPI period*
July 1–December 31, 2021	Continued normalization with residual measures	Progressive restoration of in-person education, travel, healthcare utilization, and routine social activities, while some preventive measures remained in effect	Strict-NPI period*
January 1, 2022–May 31, 2023	Progressive relaxation and withdrawal of measures	Broad restoration of routine schooling, travel, healthcare utilization, and social activities, with most remaining public health measures subsequently withdrawn	Gradual-relaxation period
